# In vitro evidence for estrogen receptor activity of selected phase II isoflavone metabolites

**DOI:** 10.1007/s00204-026-04391-2

**Published:** 2026-04-28

**Authors:** Dino Grgic, Dimitra Bella-Velidou, Luca Dellafiora, Sebastian T. Soukup, Sabine E. Kulling, Elisabeth Varga, Doris Marko

**Affiliations:** 1 Department of Food Chemistry and Toxicology, Faculty of Chemistry, University of Vienna, Währinger Str. 38-40, 1090 Vienna, Austria; 2 Doctoral School in Chemistry, University of Vienna, Währinger Str. 38-40, 1090 Vienna, Austria; 3 Department of Food and Drug, University of Parma, 43124 Parma, Italy; 4 Department of Safety and Quality of Fruit and Vegetables, Max Rubner-Institut, Federal Research Institute of Nutrition and Food, Haid-Und-Neu-Straße 9, 76131 Karlsruhe, Germany; 5 Food Hygiene and Technology, Centre for Food Science, Clinical Department for Farm Animals and Food System Transformation, University of Veterinary Medicine, Veterinärplatz 1, 1210 Vienna, Austria

**Keywords:** Estrogenicity, Phase II metabolism, Daidzein, Genistein, Glucuronides, Sulfates

## Abstract

The health benefits of soy and soy derived foods are often attributed to their isoflavone (ISFs) content, known to include phytoestrogens. However, EFSA has concluded that there is insufficient scientific evidence to establish a causal relationship between ISFs consumption and the proposed health claims. Although phase-II metabolites of ISFs are often considered as detoxified products with reduced estrogenic effects, recent studies indicate that ISFs metabolites may contribute and even enhance the biological activity of ISFs. However, current knowledge on phase-II metabolites of ISFs is limited, even though representing the major ISFs metabolites circulating in the bloodstreams in humans. This study investigates the role of phase-II metabolism in the biological activity of ISFs, focusing on estrogenicity and cytotoxicity in estrogen-sensitive Ishikawa cells (expressing estrogen receptors (ER) α and β). ISFs concentrations from 0.001 to 10 µM, with 1:10 dilution steps, were tested. Cleavage to parent compounds during incubation was also evaluated using high-performance liquid chromatography tandem mass spectrometry.

The results showed that phase-II metabolites, particularly sulfates, can still exhibit estrogenic activity, challenging the general assumption that phase-II metabolism always leads to the inactivation of ISFs. In particular, at 10 µM daidzein-4'-sulfate exhibited a comparable estrogenic activity to daidzein at the same concentration, and genistein-7-sulfate exhibited estrogenic potential at higher concentrations. Molecular dynamics simulations revealed that genistein and its conjugates can stably interact with ERβ. This study highlights the importance of phase-II metabolism for ISFs activity and suggests that the sulfates formed should be examined more closely in further investigations.

**Supplementary Information:**

The online version contains supplementary material available at 10.1007/s00204-026-04391-2.

## Introduction

**Fig. 1:**
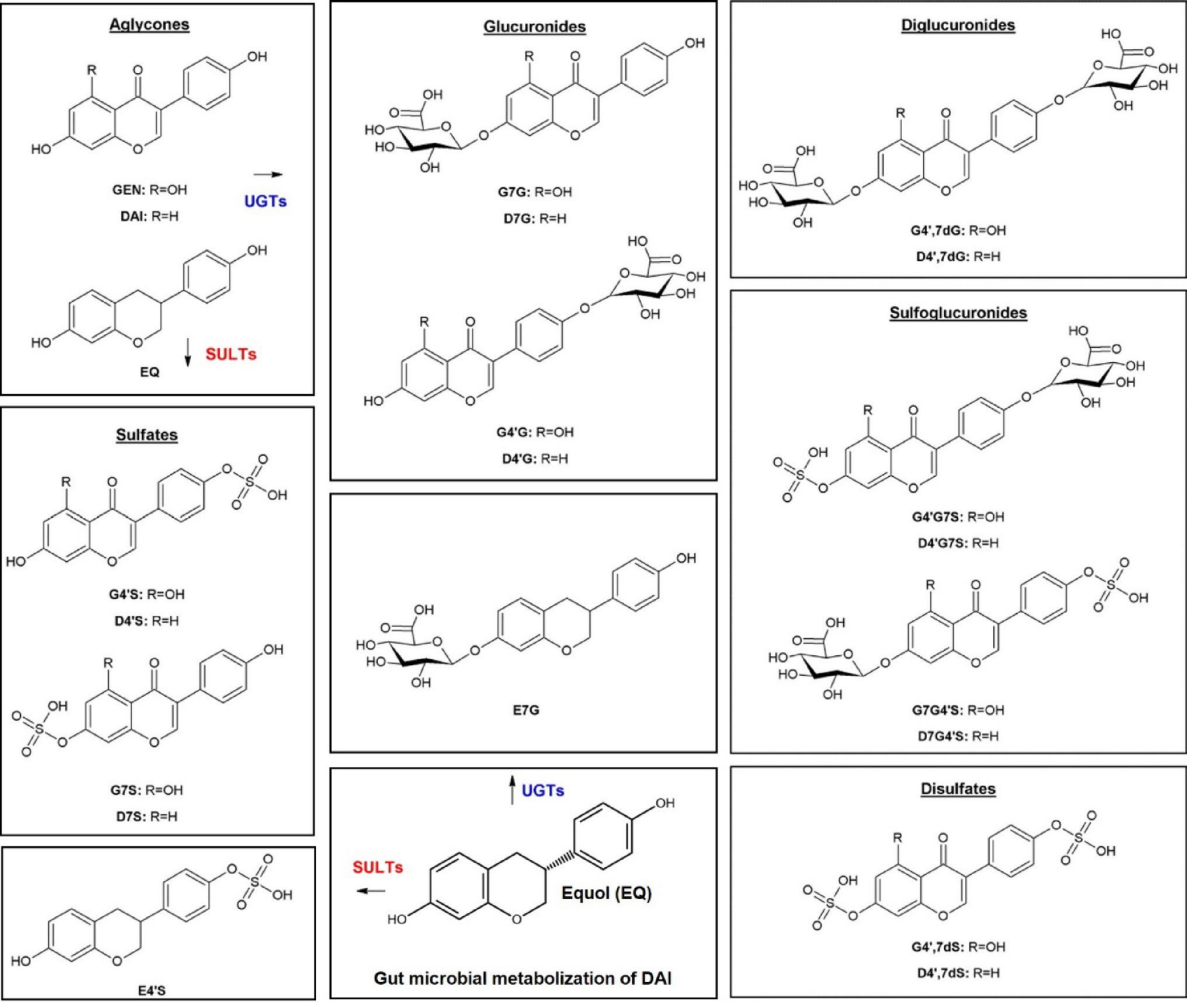
Chemical structures of the isoflavones genistein (GEN), daidzein (DAI) and the gut microbial metabolite of DAI equol (EQ) as well as selected phase II metabolites. Abbreviations: UGTs—Uridin-5'-diphospho-glucuronosyltransferase; SULTs—Sulfotransferases; G—Glucuronide, S—Sulfate

Soy and soy-based foods have often been associated with nutritional and health-promoting effects, including benefits for bone health, cardiovascular health, and alleviating menopausal symptoms. However, some reports indicate adverse effects, particularly on reproductive health. High isoflavone (ISF) intake has been linked to disruptions in the reproductive system of female farm animals, including alterations in hormonal cycles and fertility issues. These concerns primarily stem from the estrogen-like activity of ISFs, which may mimic or interfere with natural estrogen function in sensitive tissues. Additionally, long-term exposure may exacerbate these effects, raising concerns about excessive consumption in both animals and potentially humans (Hüser et al. 2018; Grgic et al. 2021). All these beneficial and adverse effects are attributed to their polyphenolic content, particularly ISFs such as genistein (GEN) and daidzein (DAI) (Fig. 1), which are secondary plant metabolites and known non-steroidal phytoestrogens (Munro et al. 2003). Beside soybeans also red clover is a potential source of ISFs. Extracts from these sources are still marketed as food supplements and other products due to their assumed positive effects on various health outcomes (Hüser et al. 2018; Chen et al. 2019). However, the European Food Safety Authority (EFSA) has so far rejected respective health claims, concluding that the scientific evidence provided was not sufficient to establish any substantial cause-and-effect relationship between ISF consumption and the aforementioned claims. As a result, advertising these health effects in relation to ISFs in food supplements and other products is prohibited (EFSA 2012).

Despite EFSA’s conclusions, research into the positive and negative effects of ISFs continues to advance globally, both in the East and West. Soy products remain popular and widely consumed, particularly with the growing adoption of vegetarian and vegan diets. EFSA has determined that daily intake of soy ISFs up to 100 mg over the duration of ten months is considered safe for healthy post-menopausal women, with no significant adverse effects on the key target organs. However, this assessment does not consider potential combinatory effects or the variability introduced by individual differences in ISFs metabolism (EFSA Panel on Food Additives and Nutrient Sources added to Food 2015). The French Agency for Food, Environmental and Occupational Health and Safety (ANSES) concluded to not recommend soy-based foods in institutional catering to avoid overconsumption (ANSES 2025).

Although phase II metabolic pathways such as glucuronidation and sulfation are generally considered as inactivation processes, studies have revealed that ISFs metabolites can contribute to and even enhance the biological activity of ISFs (Pugazhendhi et al. 2008; Beekmann et al. 2015). However, data on diconjugated phase II metabolites, namely diglucuronides, disulfates, and the glucuronide-sulfate combinations remain scarce. This is particularly concerning as analyses of human plasma samples have identified the latter as predominant metabolites (Soukup et al. 2016a, b).

To address this gap, the present study aims to provide a more detailed understanding of the role phase II metabolism plays in the biological activity of ISFs. The focus is on the ISFs GEN and DAI as well as on the microbial DAI metabolite equol (EQ), and 16 of their respective phase II metabolites, including sulfates (S), glucuronides (G), disulfates (diS), diglucuronides (diG), and sulfate-glucuronides (SG), differing in functional group positions (see Fig. 1). Estrogenicity and cytotoxicity of these individual compounds will be investigated via in vitro cell assays, complemented by high-performance liquid chromatography tandem mass spectrometry (HPLC–MS/MS) analysis to determine the profile of aforementioned substances after the incubation time. This will help clarify whether the observed effects are due to the metabolites themselves, deconjugation to the parent compounds, or further metabolization. Finally, molecular modeling will be employed to investigate the structural basis for the estrogenic activity observed in the assay.

## Materials & methods

### Materials

Cell culture 96-well plates and flasks were provided by Sarstedt (Nürnbrecht, Germany). The human endometrial adenocarcinoma cell line “Ishikawa” was obtained from the European Collection of Authenticated Cell Cultures (ECACC 99040201, Wiltshire, United Kingdom). From Gibco, Thermo Fisher Scientific (Waltham/MA, USA) Minimal Essential Medium (MEM) and Dulbecco’s Modified Eagle Medium/F12 (DMEM/F-12) without phenol red, supplemented with fetal bovine serum (FBS), charcoal-stripped FBS (CD-FBS), L-glutamine and penicillin–streptomycin (P/S) were obtained. Sigma Aldrich Chemie GmbH (Schnelldorf, Germany) provided 17β-estradiol (E2), 4-nitrophenylphosphate, diethanolamine, MgCl_2_, and sulforhodamine B (SRB). The aglycons and 16 of their phase II metabolites were acquired at various companies or synthesized ourself (unless otherwise stated, purities were determined by us using HPLC–DAD): daidzein (DAI) (purity 99.2%), R,S-equol (EQ) (99.3%) and genistein (GEN) (> 99.9%) all from LC Laboratories (Woburn/MA, USA); (R,S)-equol-4’-sulfate (E4’S) (90%, stated by manufacturer), (R,S)-equol-7-glucuronide (E7G) (98%, stated by manufacturer) all from Santa Cruz Biotechnology Inc.; daidzein-4’,7-disulfate (D4’,7dS) (99.5%) and genistein-4’,7-disulfate (G4’,7dS) (99.4%) were synthesized as described in (Soukup et al. 2014) daidzein-4’,7-diglucuronide (D4’,7dG) (93%), daidzein-4’-glucuronide-7-sulfate (D4’G7S) (98.7%), daidzein-4’-sulfate (D4’S) (95%, stated by manufacturer), daidzein-7-glucuronide (D7G) (99.2%), daidzein-7-glucuronide-4’-sulfate (D7G4’S) (> 99.9%), daidzein-4’-glucuronide (D4’G) (94.4%), genistein-4’,7-diglucuronide (G4’,7dG) (90.8%), genistein-4’-glucuronide (G4’G) (99%), genistein-4’-glucuronide-7-sulfate (G4’G7S) (98.9%), genistein-7-glucuronide (G7G) (95%, stated by manufacturer), genistein-7-glucuronide-4’-sulfate (G7G4’S) (99%), genistein-7-sulfate (G7S) (97%, stated by manufacturer) all from Toronto Research Chemical Inc. (Toronto/ON, CA). Roth (Karlsruhe, Germany) provided dimethly sulfoxide (DMSO), NaCl, KCl, Na_2_HPO_4_ × 2 H_2_O and KH_2_PO_4_. Phosphate-buffered saline PBS (10x) was being prepared by dissolving 1.71 M of NaCl, 100 mM of Na_2_HPO_4_, 34 mM of KCl and 18 mM of KH_2_PO_4_ in distilled water, the pH-value was adjusted to 7.4, the solution sterile filtered and stored at 4 °C until further usage. PBS (1x) was prepared by diluting PBS (10x) 1:10 with distilled water. The CellTiter-Blue^®^ Cell Viability Assay Kit was purchased from Promega Corporation (Madison/WI, USA). Acetonitrile (ACN) and methanol (MeOH) were of CHROMASOLV^TM^ LC–MS quality and obtained from Honeywell Riedel-de Haën™ (Seelze, Germany).

### Cell line

Ishikawa cells were stored in liquid nitrogen containers, and two weeks prior to the start of the in vitro experiments, they were cultured using MEM supplemented with 5% (*v/v*) heat-inactivated FBS, 1% L-glutamine and 1% P/S. Cells were grown in an incubator at 37 °C with 5% CO_2_ and 95% humidity, split at approximately 80% confluency, and maintained up to a maximum passage number of 40. Before conducting the assays, the growth medium was replaced with the assay medium consisting of DMEM/F-12 supplemented with 5% CD-FBS and 1% P/S.

### Alkaline phosphatase assay

To perform the assay, 15,000 or 10,000 Ishikawa cells in assay medium were seeded in each of the inner wells of a 96-well plate, allowing the cells to adhere and grow for 24 or 48 h, respectively. Incubation was then carried out with different concentrations of GEN, DAI, EQ, and 16 of their phase II metabolites as individual substances, at different concentrations. These substances were initially dissolved in DMSO at 200 times the tested concentration and subsequently diluted in the assay medium. DMSO was added to reach 1% in the final incubation solutions for single substance testing and the controls. The tested concentration range was 0.001 to 10 µM, with 1:10 dilution steps in between. E2 (1 nM) served as positive control. All experiments were conducted in at least five independent biological replicates (measurements with different cell passages) with technical triplicates (repeated measurements with the same cell passage) each. Following the 48-h incubation, the supernatants were discarded, and the wells were washed three times with 150 µL PBS (1x) per well. After removing the PBS completely, the plate was placed in the freezer at − 80 °C for at least 20 min, which resulted in cell lysis and the release of ALP. The plate was then thawed for five minutes at room temperature before 50 µL of ALP buffer (containing 5 mM 4-nitrophenylphosphate, 1 M diethanolamine, and 0.24 mM MgCl_2_) was added in the dark. After five minutes at room temperature, the plate was placed in a plate reader, and absorbance was measured at 405 nm every two minutes for one hour at 37 °C using either a Victor V3 1240 Multilabel Counter from Perkin Elmer (Waltham/MA, USA) or a Cytation 3 Cell Imaging Multi-Mode Reader from Biotek^®^ (Winooski/VM, USA). The ALP activity was calculated as the slope of the curve obtained by the measurements monitored over one hour. Final results were referred to the solvent (1% DMSO) and positive control (1 nM E2), which were set to 0 and 100%, respectively. The data obtained from the experiments were analyzed statistically (see separate section) to determine the effect of each ISFs on ALP activity in Ishikawa cells.

### Coupled CellTiter-Blue^®^ and SRB cytotoxicity assays

After seeding 15,000 or 10,000 cells per well and allowing them to grow for 24 or 48 h, respectively, the cells were exposed to incubation solutions containing individual substances, as described for the ALP assay. A 1% DMSO solution was used as the solvent control. The experiments were performed in five independent biological replicates, each with technical triplicates. After 48 h of incubation, the medium was aspirated, and 100 µL CellTiter-Blue^®^ (CTB) incubation solution (1:10 dilution of CTB reagent in DMEM/F-12 (5% CD-FBS, 1% P/S)) was added to each well. After incubating for another 50 min, 90 µL of each well were transferred to a new, black 96-well plate and measured at an excitation wavelength of 560 nm and an emission of 590 nm using one of the plate readers specified above in the ALP-section. The results were calculated as a percentage of the solvent control.

The remaining 10 µL of the initial 96-well plate were aspirated, and the cells were fixed with 10 µL of a 50% (*w/v*) trichloroacetic acid solution in distilled water for one hour at 4 °C. The plate was then washed four times with tap water and dried overnight in the dark. Afterwards, 50 µL of the SRB solution (4 g SRB reagent solved in 1 L distilled water containing 1% acetic acid) were added to each well. After one-hour staining in the dark at room temperature, the coloring solution was discarded, and the plate was washed twice with tap water and twice with 1% acetic acid. After another overnight drying step in the dark, the dye was dissolved under alkaline conditions in 100 µL Tris base (0.30 g tris(hydroxymethyl-) aminomethane solved in 250 mL distilled water) by shaking for 5 min in the plate reader (Victor V3 1240 Multilabel Counter from Perkin Elmer (Waltham/MA, USA) or the Cytation 3 Cell Imaging Multi-Mode Reader from Biotek^®^ (Winooski/VT Vermont, USA)). Subsequently, the absorbance was measured at 570 nm. The results were calculated as a percentage of the solvent control, as for the CTB assay.

### Chemical analysis

The cell cultivation, seeding of the cells and the incubation with the test substances was performed as already described in the previous section of the ALP assay. The only exception was that estrogenic compounds were prepared in concentrations of 1 μM and 10 μM, while non-estrogenic compounds were prepared only at the highest concentration of 10 μM. Cells were also prepared with no test substance and solvent control (1% DMSO). Four biological replicates (measurements with different cell passages), each consisting of two technical replicates (repeated measurements with the same cell passage) were prepared to ensure reproducibility of the results, and after 48 h of incubation, 80 μL supernatant was transferred to microtubes containing ice cold ACN, resulting in a 1:2 dilution, and stored at -80 °C until further processing. The solutions were thawed, centrifuged (10 min at 4 °C and 18,620 rcf) and 100 μL of supernatant was transferred to glass HPLC vials with microinserts for measurement and 2 µL thereof was injected. The samples were analyzed using a 1260 Infinity II LC System (Agilent Technologies, Santa Clara/CA, USA) coupled to a QTRAP^®^ 6500+ from Sciex (Framingham/MA, USA). The developed high-performance liquid chromatographic tandem mass spectrometric (HPLC–MS/MS) method was based on Soukup *et. al.* (Soukup et al. 2014). Separation was performed on a Supelco Ascentis^®^ Express C18 column (100 × 2.1 mm, 2.7 μm) and a C18 guard column (Phenomenex, Aschaffenburg, Germany) with 10 mM ammonium carbonate in water and ACN/MeOH (1/2.5) as eluents A and B. The autosampler temperature was set to 5 °C, and the column temperature was 40 °C. The flow rate was 0.5 mL/min and the applied gradient was as follows: 0–2.6 min: 3% B, 2.6–10.7 min: linear increase to 56% B, 10.7–11.0 min: linear increase to 95% B, 11.0–14.0 min: holding period at 95% B, 14.0–14.1 min: decrease to 3% B and column equilibration until 16.5 min. Data were acquired in multiple reaction monitoring (MRM) mode using negative electrospray ionization and specific transitions are provided in Supplement Table S1. All compounds were previously optimized using syringe injection of ACN-stock solutions (100 mg/L) diluted with eluent A:B (1:1, *v/v*). Qualitative data evaluation was performed with Analyst (v.1.7.) and Sciex OS (v. 3.1.0.16485). External calibration standards were prepared from individual stock solutions (100 mg/L in ACN) by first preparing a master-mix which was further diluted in serial dilutions with (MeOH:H_2_O, 30:70) to obtain concentrations between 0.1 and 3000 µg/L. Quantitative analytes were performed with Skyline (v. 22.2; MacCoss Lab, Department of Genome Sciences, University of Washington, Seattle/WA, USA).

To check whether the mass spectrometric results are significantly influenced by matrix effects and to calculate the recovery rates, four different spiking solutions were prepared. These solutions consisted of i) the three IFS or the metabolites of ii) GEN, iii) DAI or iv) EQ. For the spiking procedure a biological replicate of the solvent control (1% DMSO) treatment after 48 h of incubation was used. For recovery calculation an aliquot (80 μL) of the supernatant was spiked with 10 µL of spiking solution prior to adding 70 µL ice-cold ACN (solution #1). For the assessment of matrix effects an aliquot (80 μL) of the supernatant was treated with 80 µL ice-cold ACN (solution #2). Both experiments were performed in triplicate. All solutions were then stored at -80 °C until further processing. After more than 72 h, the test samples were processed as described above for the samples. After centrifugation, 140 μL of the supernatant of solution #1 was transferred to HPLC vials containing microinserts. In case of solution #2, 140 μL were spiked with 10 μL of the aforementioned spiking solutions. The theoretical values were compared with the calculated values obtained with the external calibration functions.

### Molecular modeling in silico

The in silico analysis consisted in an integrated use of docking studies, to predict the binding architecture between the polyphenols under analysis and the ERβ, and molecular dynamics simulations to study the polyphenol-ERβ complex stability over time. The 3D structure of polyphenols has been retrieved in the.sdf format from PubChem (https://pubchem.ncbi.nlm.nih.gov) (Kim et al. 2023) with the following PubChem ID: GEN 5280961; G7S 10291508; G7G 15940724; G4’G 45782816; G4′7dS 44602471; G7G4’S 54020950; G4′7dG 101519310; G4’G7S 102339163; DAI 5281708; D4’S 12114463; D4’G 23930394; D4’,7dS 54484332; D7G 11316354; D7G4’S 23643386; D4’,7dG 101098147; D4’G7S 156960880; EQ 91469; E7G 29979359 and E4’S 29979373. The model for ERα and ERβ has been derived from the Protein Data Bank structure with ID 5KRC, as previously described (Dellafiora et al. 2020) and 1X7J (Manas et al. 2004) respectively, in agreement with previous studies (Dellafiora et al. 2020). Docking studies have been performed in agreement with previous studies (Dellafiora et al. 2015) with GOLD (version 2021.1). Briefly, the internal GOLDScore scoring function was used, the binding site was set within a 5 Å radius sphere around the centroid of the co-crystallized ligand-binding site. Also, a semi-flexible docking protocol was applied while allowing protein’s polar hydrogens free to rotate and considering ligands fully flexible. Molecular dynamics (MD) simulations were performed through GROMACS (version 2021.4) (Abraham et al. 2015) to monitor the geometrical stability of the complex and ligand orientation. All the ligands were processed and parametrized with the SwissParam tool (https://ww.swissparam.ch) (Zoete et al. 2012) and the whole system was parametrized with the CHARMM27 all-atom force field (Brooks et al. 2012). The input complex structures were solvated with SPCE water in a dodecahedron periodic boundary condition and neutralized adding Na^+^ and Cl^-^ as counter ions. Before running MD simulations, each system underwent an energetical minimization to both avoid steric clashes and correct improper geometries using the steepest algorithm with a maximum of 5000 steps. Then, each system underwent isothermal (300 K; coupling time of 2 ps) and isobaric (1 bar; coupling time of 2 ps) 100 ps simulations before undergoing 25 ns long MD simulations (300 K with a coupling time of 0.1 ps and 1 bar with a coupling time of 2 ps).

### Statistics

The ALP and cytotoxicity assays were conducted with technical triplicates and at least five independent biological replicates (n ≥ 5). Outliers were identified using the Nalimov and Kolmogorov–Smirnov tests for normality and excluded from the calculation of mean values and standard deviations. At most, one outlier was excluded, resulting in at least four biological replicates. Origin Pro^®^ 2021 software was used for statistical analyses and data plotting, with significance levels of 5%, 1%, and 0.1% denoted by * = p < 0.05; ** = p < 0.01; and *** = p < 0.001, respectively. Significant differences were evaluated using one-way analysis of variance (ANOVA) followed by Fisher’s least significant difference (LSD) post-hoc test. Cytotoxicity results were assessed using one-way and two-way Student’s *t*-tests.

## Results

### Alkaline phosphatase assay (ALP)

**Fig. 2:**
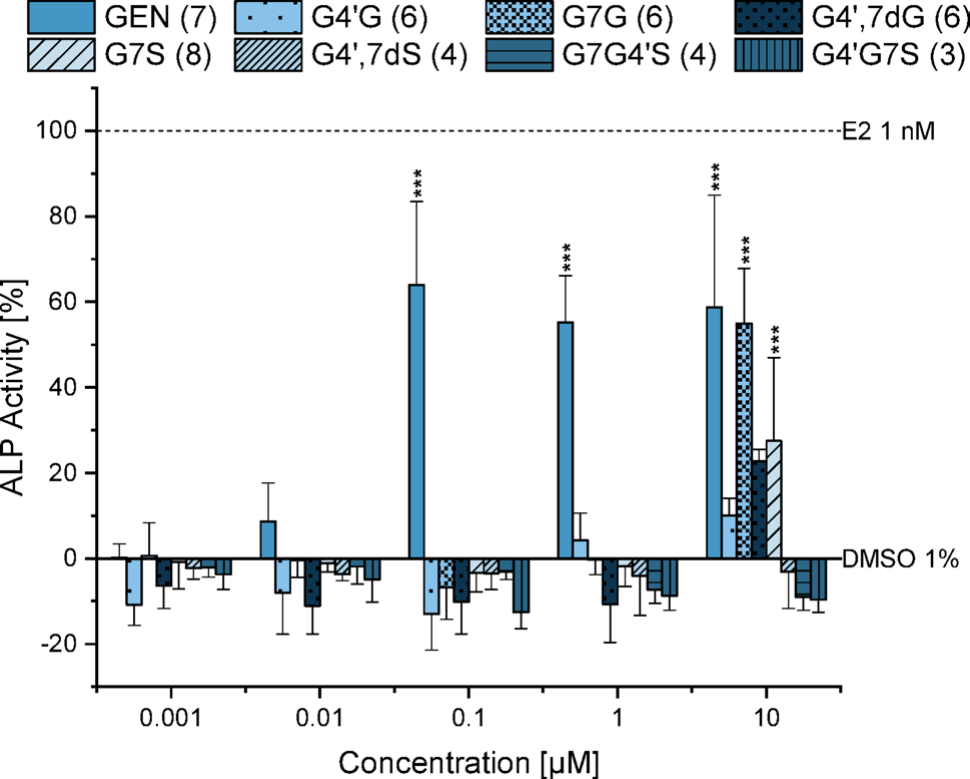
Graphic depiction of the estrogenic induction by genistein (GEN) and seven of its metabolites in Ishikawa cells. The ALP activity of genistein (GEN), genistein-4’,7-disulfate (G4’,7dS), genistein-4’,7-diglucuronide (G4’,7dG), genistein-4’-glucuronide (G4’G), genistein-4’-glucuronide-7-sulfate (G4’G7S), genistein-7-glucuronide (G7G), genistein-7-glucuronide-4’-sulfate (G7G4’S), genistein-7-sulfate (G7S) [%] after 48 h of incubation is shown across the concentration range [0.001 – 10 μM], with DMSO (1%) as solvent control and 17β-estradiol (1 nM) as positive control representing 0 and 100% respectively. Results are depicted as mean ± standard deviation values in bar form. The number of biological replicates for each substance (ranging from 3 to 8) is indicated in parentheses next to the abbreviated names in the graph legend. Outliers identified by the Nalimov outlier test were excluded. Significant differences of effects between solvent control and the incubated compounds were calculated using a one-sample Student’s *t*-test and are marked with *** (p < 0.001).

The results for GEN and seven of its phase II metabolites are depicted in Fig. 2. At concentrations of 0.001 and 0.01 μM, neither GEN nor its metabolites showed significant estrogenic activity. GEN displayed no estrogenic activity at 0.01 µM (9 ± 9%), with a significant increase at 0.1 μM (64 ± 19%), followed by 55 ± 11% at 1 μM and a plateau at 59 ± 26% at 10 μM, consistent with previous findings (Grgic et al. 2022). Neither the 4’-O-glucuronide nor the 7-O-glucuronide or the diglucuronide showed estrogenic activity at concentrations of 0.001 to 1 μM. The combined glucuronide-sulfate metabolites did not exhibit estrogenic activity at any of the tested concentrations. However, at the highest concentration of 10 µM, the monoglucuronide (G4’G, G7G), diglucuronide (G4’,7dG) and the sulfate (G7S) of GEN were able to induce ALP activity. For the 7-O-glucuronide and the 7-O-sulfate the impact on ALP were even significantly increased compared to the solvent control.

**Fig. 3:**
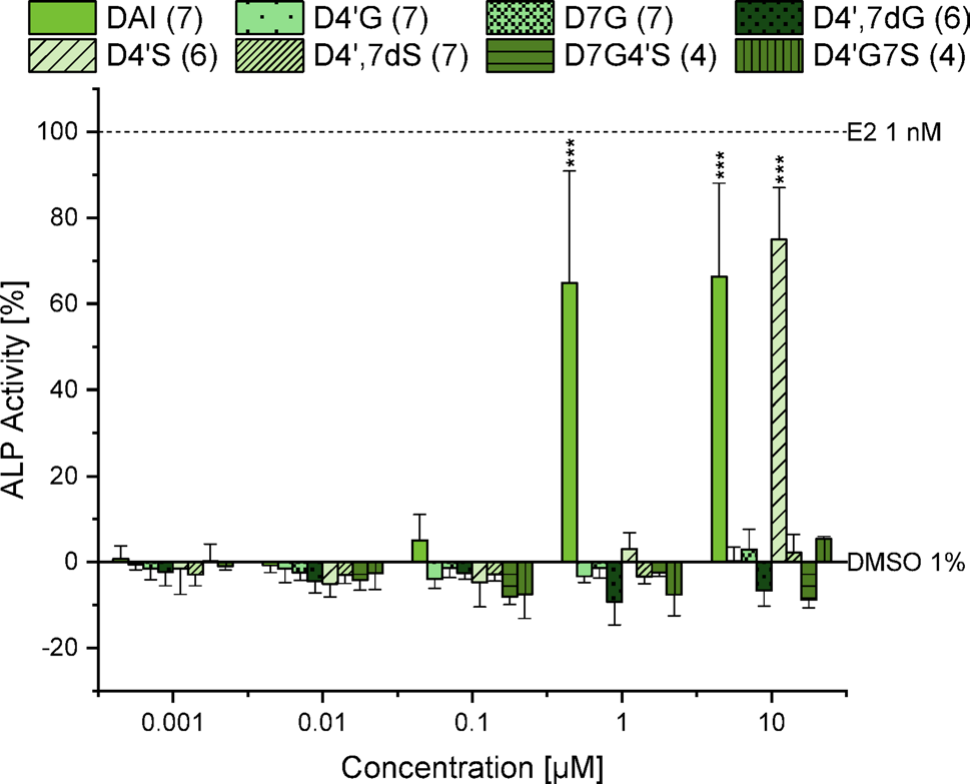
Graphic depiction of the estrogenic induction by daidzein (DAI) and seven of its metabolites in Ishikawa cells. The ALP activity of daidzein (DAI), daidzein-4’,7-disulfate (D4’,7dS), daidzein-4’,7-diglucuronide (D4’,7dG), daidzein-4’-glucuronide-7-sulfate (D4’G7S), daidzein-4’-sulfate (D4’S), daidzein-7-glucuronide (D7G), daidzein-7-glucuronide-4’-sulfate (D7G4’S), daidzein-4’-glucuronide (D4’G) [%] after 48 h incubation is provided against the concentration [0.001 – 10 μM], with DMSO (1%) as solvent control and 17β-estradiol (1 nM) as positive control representing 0 and 100% respectively. Results are depicted as bars of mean + or – standard deviation values. The biological replicates of each substance (ranging from 4 to 7) are shown in the parentheses next to the abbreviated names in the legend above the graph. Outliers after Nalimov outlier test were excluded. Significant differences of effects between solvent control and the incubated compounds were calculated by one-sample Student’s *t*-test and were indicated with *** (p < 0.001)

In these experiments, the tested concentrations of DAI-metabolites had varying effects on ALP activity (Fig. 3). Concentrations of DAI and its seven metabolites ranging from 0.001 to 0.1 μM showed no estrogenic activity, with the exception of weak activity (5 ± 6%) observed for DAI at 0.1 μM. However, at a concentration of 1 μM, a significant increase in ALP activity was observed for DAI, with a stable activity of 65 ± 26% maintained at the highest concentration of 10 μM. None of the glucuronides or the diglucuronide metabolites of DAI induced estrogenicity in the tested concentrations, and the same was observed for sulfoglucuronide combinations, D7G4’S, and D4’G7S. At 10 µM D4’S exhibited a comparable estrogenic activity to DAI at 1 and 10 μM, while no significant estrogenicity was observed below this concentration.

**Fig. 4:**
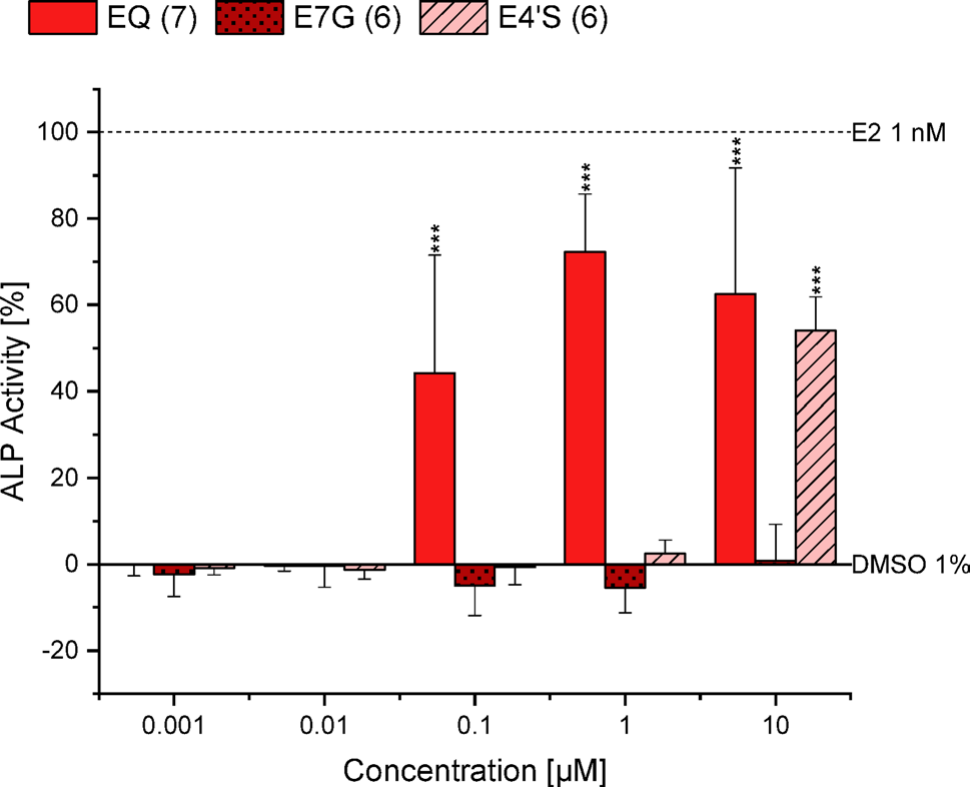
Graphic depiction of the estrogenic induction by equol (EQ) and two of its metabolites in Ishikawa cells. The ﻿ALP activity of (S)-equol, (EQ), (S)-equol-4’-sulfate (E4’S), (S)-equol-7-glucuronide (E7G) [%] after 48 h incubation is provided against the concentration [0.001 – 10 μM], with DMSO (1%) as solvent control and 17β-estradiol (1 nM) as positive control representing 0 and 100% respectively. Results are depicted as bars of mean + or – standard deviation values. The biological replicates of each substance (ranging from 6 to 7) are shown in the parentheses next to the abbreviated names in the legend above the graph. Outliers after Nalimov outlier test were excluded. Significant differences of effects between solvent control and the incubated compounds were calculated by one-sample Student’s *t*-test and were indicated with *** (p < 0.001)

None of EQ metabolites induced changes in ALP activity up to a concentration of 0.1 μM, where the aglycone exhibited a 44 ± 27% increase compared to the solvent control (Fig. 4). At a concentration of 1 μM, a higher level of estrogenicity was observed, reaching 77 ± 13%, followed by 63 ± 29% at the highest concentration at 10 μM. At the highest concentrations (10 µM) E4’S was able to induce ALP activity which was comparable to that of its aglycone at 0.1 µM and above.

### Cytotoxicity

**Fig. 5:**
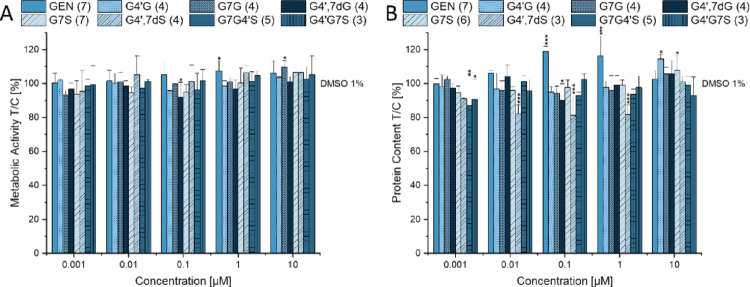
Graphic depiction of the effects of genistein (GEN) and its metabolites on Ishikawa cells. The ﻿cell viability after 48 h incubation is provided as metabolic activity [%] measured by the CTB assay (A) and as cell protein content [%] measured by the SRB assay (B) against the concentrations of the substances [0.001 – 10 μM]. The values were referred to DMSO (1%) as solvent control represented as 100%. Results are depicted as bars of mean + standard deviation values. The biological replicates of each substance (ranging from 3 to 7) are shown in the parentheses next to the abbreviated names in the legend above the graphs. Outliers after Nalimov outlier test were excluded. Significant differences of effects between solvent control and the incubated compounds were calculated by one-sample Student’s *t*-test and were indicated with * (p < 0.05), ** (p < 0.01) and *** (p < 0.001)

In general, only minimal changes in cytotoxicity were observed based on the CTB data (Fig. 5A). The metabolic activity of most compounds was comparable to that of the solvent control. However, a slight reduction in viability was noted at the lowest concentration of G7G, G7S, and G4′7dS. The least metabolic ability recorded in all GEN data sets was for G4′7dG, which displayed a viability of 92 ± 9% at a concentration of 0.1 μM.

Interestingly, the SRB data revealed more pronounced effects (Fig. 5B). GEN exhibited proliferative activity at concentrations of 0.1 μM and 1 μM, with protein content of 119 ± 3% and 116 ± 12%, respectively, which decreased at 10 μM. The highest concentration of G4’G also led to increased proliferation, with a protein content of 115 ± 2%. In contrast, the most noticeable decrease in cell viability was observed for G4’,7dS at concentrations of 0.01 μM, 0.1 μM, and 1 μM, each resulting in approximately 82% viability. Similar results were obtained for the lowest concentrations of sulfoglucuronides.

**Fig. 6:**
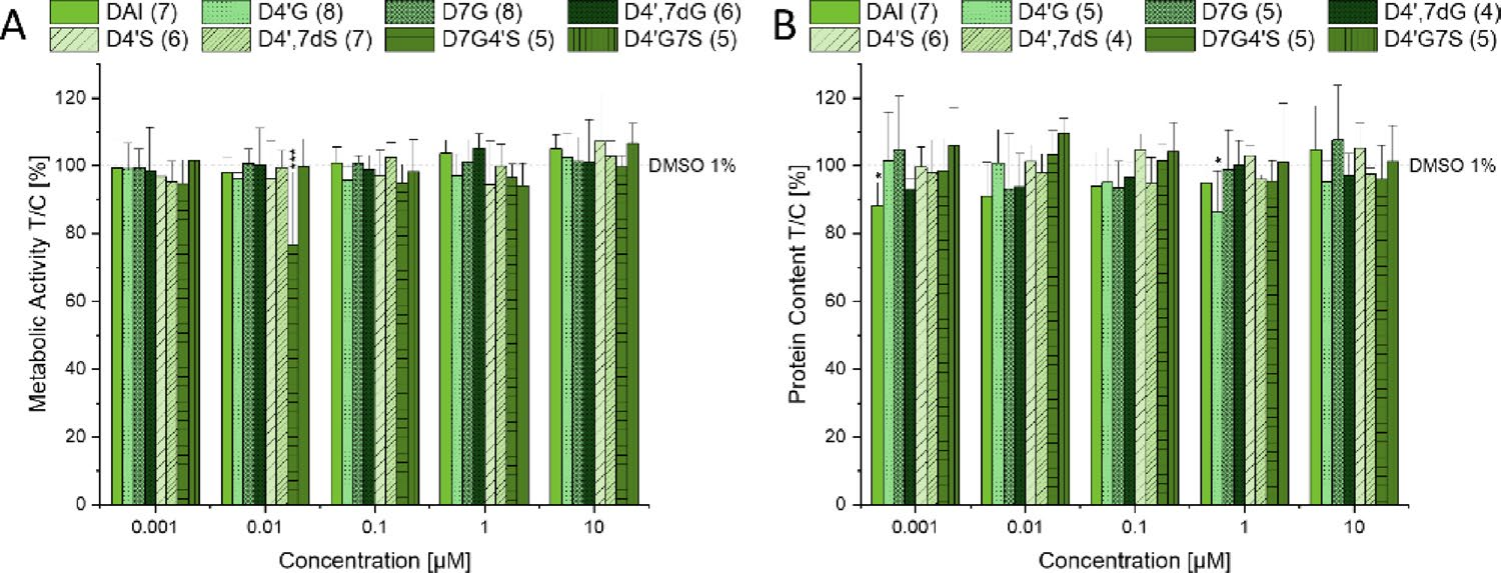
Graphic depiction of the effects of daidzein (DAI) and its metabolites on Ishikawa cells. The cell viability after 48 h incubation is provided as metabolic activity [%] measured by CTB assay (A) and as cell protein content [%] measured by SRB assay (B) against the concentrations of the substances [0.001 – 10 μM]. The values were referred to DMSO (1%) as solvent control represented as 100%. Results are depicted as bars of mean + standard deviation values. The biological replicates of each substance (ranging from 4—8) are shown in the parentheses next to the abbreviated names in the legend above the graphs. Outliers after Nalimov outlier test were excluded. Significant differences of effects between solvent control and the incubated compounds were calculated by one-sample Student’s *t*-test and were indicated with * (p < 0.05), ** (p < 0.01) and *** (p < 0.001)

There are far fewer observations available for DAI and its metabolites (Fig. 6). In contrast to GEN, DAI did not exhibit any significant proliferation in terms of metabolic activity, although a slight increase was observed at higher concentrations, which was not significant. Apart from the sulfoglucuronide D7G4’S, which showed a trend to anti-proliferative effects at concentrations ranging from 0.001 μM to 1 μM, no other effects were observed in the CTB data. The minimum anti-proliferative effect was observed at 0.01 μM with 77 ± 22% viability, although the standard deviation for this particular case was high, and this effect was not seen in the other sulfoglucuronide. Similar to GEN, anti-proliferative effects were only observed in the SRB data for DAI (Fig. 6B). Contrary to the CTB results, DAI resulted in decreased cellular protein content for all concentrations except the highest one, although significant difference was only observed at 0.001 µM. Additionally, slight mostly non-significant anti-proliferative effects were exhibited for D4’G (0.1 μM to 10 μM), D7G (0.01 μM to 0.1 μM), and for D4’,7dG concentrations ranging from 0.001 μM to 0.1 μM.

**Fig. 7:**
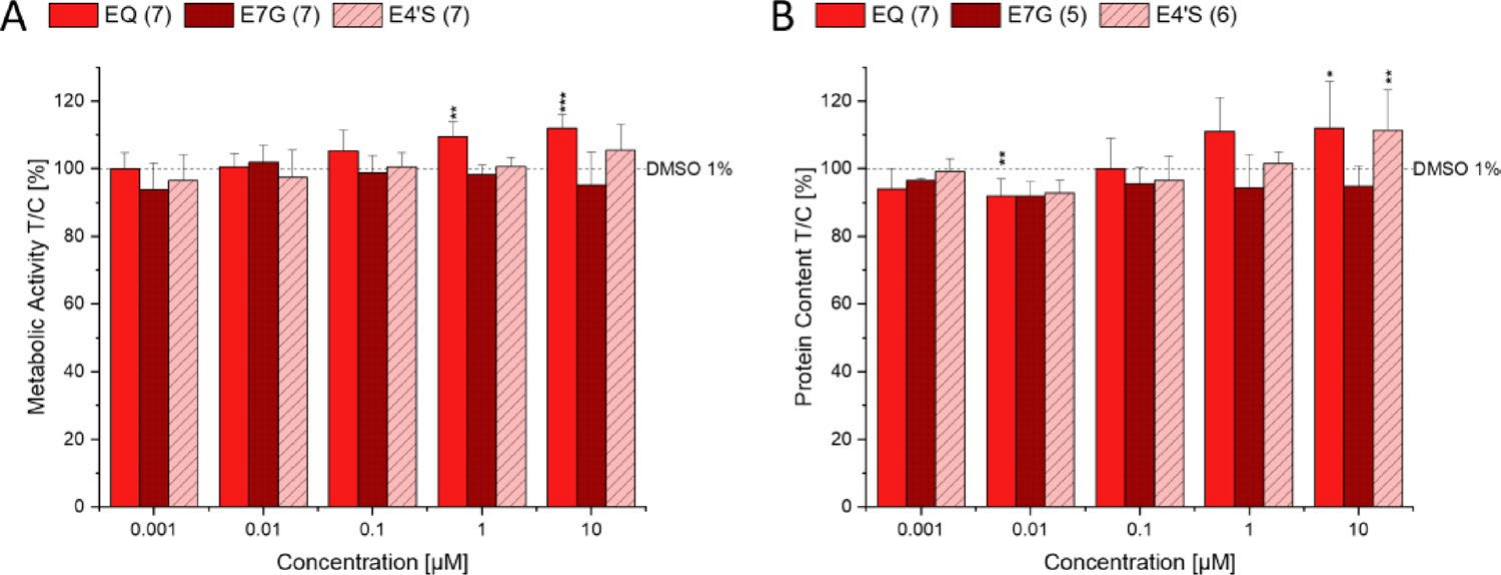
Graphic depiction of the effects of equol (EQ) and its metabolites on Ishikawa cells. The cell viability after 48 h incubation is provided as metabolic activity [%] measured by CTB assay (A) and as cell protein content [%] measured by SRB assay (B) against the concentrations of the substances [0.001 – 10 μM]. The values were referred to DMSO (1%) as solvent control represented 100%. Results are depicted as bars of mean + standard deviation values. The biological replicates of each substance (ranging from 5 to 7) are shown in the parentheses next to the abbreviated names in the legend above the graphs. Outliers after Nalimov outlier test were excluded. Significant differences of effects between solvent control and the incubated compounds were calculated by one-sample Student’s *t-*test and were indicated with * (p < 0.05), ** (p < 0.01) and *** (p < 0.001)

In both assays, the phytoestrogen gut microbial metabolite EQ showed a weak concentration-dependent increase in cell proliferation (Fig. 7). Notably, in CTB, the increase was gradual and continued until it reached a metabolic activity of 112 ± 4% for the highest tested concentration. In SRB, although there was a similar trend for concentrations ranging from 0.1–10 μM, there was a slight but insignificant decrease in proliferation observed at the lowest concentrations, reaching a minimum protein content of 92 ± 5%.

.

### Chemical analysis

**Fig. 8:**
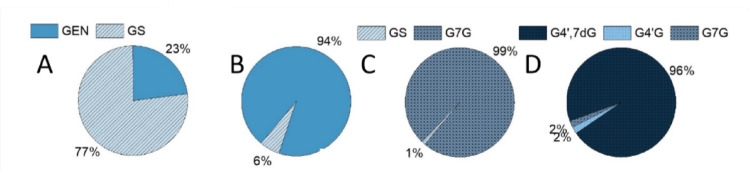
Pie charts representing the metabolites present in the cell supernatant after incubating Ishikawa cells for 48 h with GEN **A**—1 µM; **B**—10 µM) or its metabolites **C**—1 µM G7G and **D**—10 µM G4’,7dG

After a 48-h incubation of the individual ISFs metabolites with Ishikawa cells the cell supernatant was analyzed. The most significant changes occurred for the ISFs themselves, with notable differences observed in metabolization between a concentration of 1 μM and 10 μM of GEN (Fig. 8). After 48 h of incubation, 77% of GEN was metabolized to a sulfate counterpart at 1 μM, while only 6% of the sulfate was formed at 10 µM (Fig. 8A/B). Regarding the studied conjugates of GEN, there were only two cases in which minor detectable components were found in addition to the respective parent compound: G7G at 1 μM (Fig. 8C) and G4’,7dG at 10 μM (Fig. 8D). In the samples of the former one 1% GS was detectable, while for the latter one the both mono-glucuronides with a 4% yield in total were measurable. The other studied conjugates of GEN including G4’,7dS, G7S, G4’G7S, G7G4’S, G4’G, and G7G (at 10 μM), were neither metabolized by the Ishikawa cell line nor converted back to the aglycone.

**Fig. 9:**
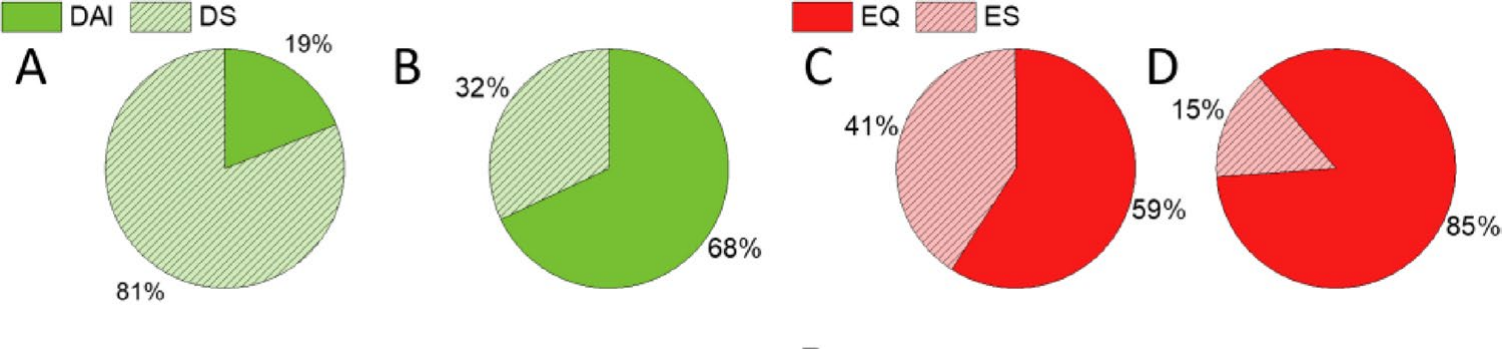
Pie charts representing the metabolites present after incubating Ishikawa cells for 48 h with DAI **A**—1 µM, **B**—10 µM or EQ **C**—1 µM, **D**—10 µM

Similar to GEN, DAI also undergoes sulfation to form its sulfate counterpart (Fig. 9A, B). At the concentration of 1 µM, 81% of DAI was converted into a sulfate metabolite, while the highest concentration of 10 µM resulted in a lower percentage rate of sulfate, with only 32% sulfates and 68% DAI remaining. The pattern of EQ metabolism was similar to that of GEN and DAI, with the aglycone being converted to a monosulfate (Fig. 9C, D). At 1 μM, 41% of the monosulfate was detected in the samples, which decreased to 15% at a higher concentration of 10 µM. Unlike GEN and DAI, the majority of the molecule remained in its aglycone form at both tested concentrations.

Regarding the DAI metabolites, the vast majority of the compounds remained unchanged, with few cases in which minor components (< 3%) were detectable (see supplementary Fig. S1 for graphical representation). In case of the diglucuronide, 1% of a monoglucuronide was detected. Furthermore, for the disulfate a monosulfate was measurable, with a percentage of 3%.

**Fig. 10:**
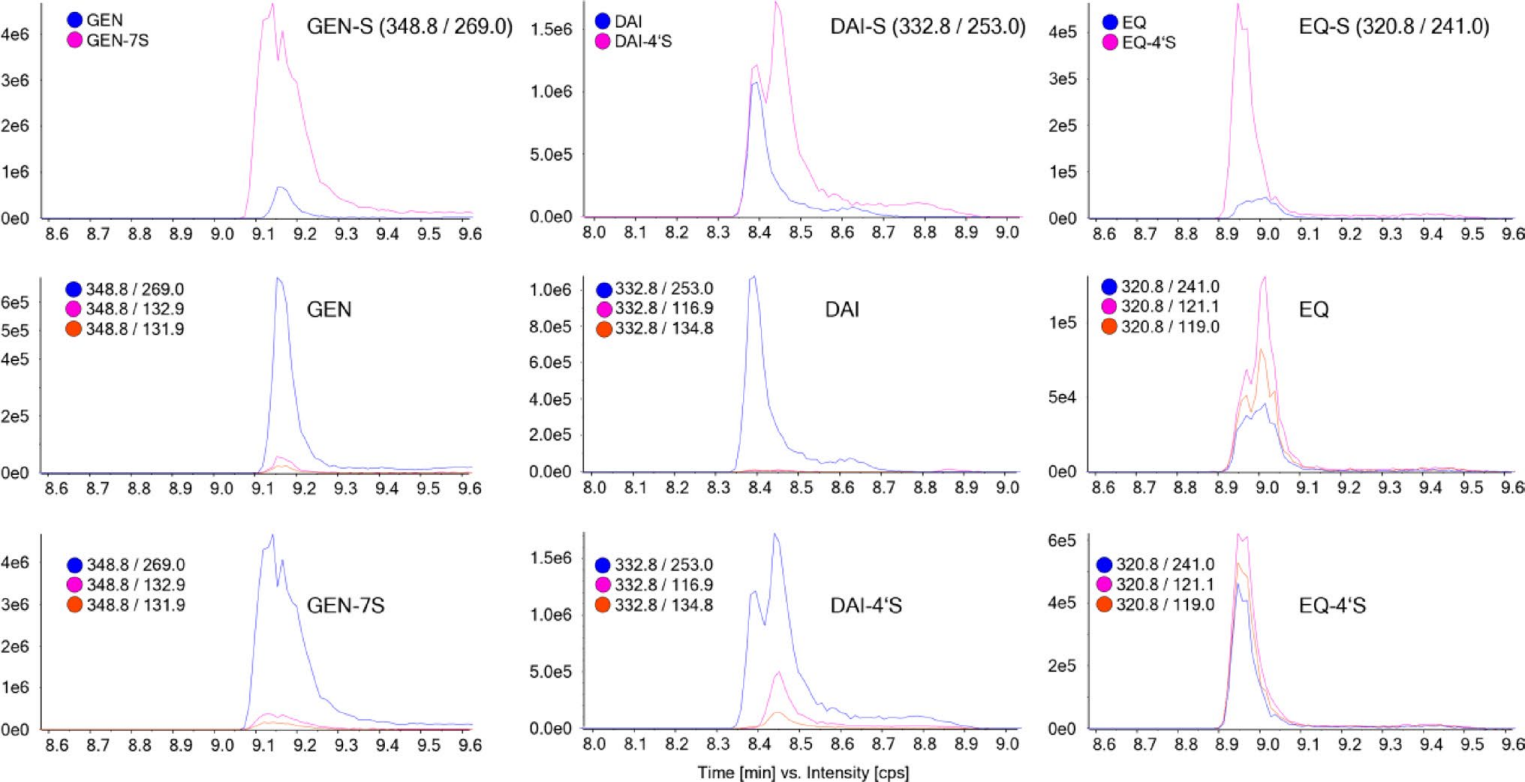
Extracted ion chromatograms showing an overlay of the most intensive transition of an aglycon and a sulfate sample (top) and an overlay of all three measured transitions of the aglycon (middle) and sulfate (bottom) sample separately for the three isoflavones (GEN—genistein, DAI—daidzein, EQ—S-equol) investigated

It is important to point out that only one sulfate-conjugate was available for each isoflavone (GEN-7S, DAI-4’S and EQ-4’S). Furthermore, chemical analysis revealed that the DAI-4’S standard was not pure and most probably contaminated with DAI-7S since a double peak was visible (Fig. 10). The pattern matched that observed after incubating the presumed DAI-7S for 48 h, indicating no changes over the incubation period. Therefore, and due to the lack of baseline separation between the two isoforms (4’ and 7-sulfate), sum parameters were calculated and are referred to as GS, DS and ES for the sulfates of GEN, DAI and EQ, respectively. In case of GEN and EQ no clear split peaks were visible, but the presence of two overlapping isoforms cannot be entirely ruled out. Figure 10 provides an overview of the sulfate transitions.

Spiking experiments revealed recovery and matrix effects values between 80 and 110%, with a few exceptions (Supplementary Table S2). No corrections of the results were made since with the exception of the isoflavones no significant changes during the incubation period took place. In case of D4’,7dG, a spiking level that was too low was chosen, and the resulting peaks were below the limit of detection. Since no significant changes were observed for this metabolite after the 48 h of incubation, this experiment was not repeated with higher concentrations.

### Molecular modeling

First, docking studies have been performed to propose plausible binding architectures for the ISFs under analysis with ERα and ERβ, and to evaluate their respective fitness within the ERs’ ligand pockets (with higher scores indicating better fitting into the pocket), consistent with previous studies (Dellafiora et al. 2015) and aligning with manufacturer declarations (https://www.ccdc.cam.ac.uk). Then, molecular dynamics simulations were performed on a selection of ISFs to check the stability of the ISFs-ERβ complex over time.

As shown in Table 1, the conjugation of GEN, DAI, and EQ was associated with a decrease in docking scores compared to their respective parental compounds suggesting a reduced capability to fit into the ERs pockets. Despite reduced affinity compared to their aglycones, ISFs conjugated with monosulfates still demonstrated positive and relatively high scores, suggesting a binding potential within ER pockets.

**Table 1:** Docking results expressed as GOLDScore units of the isoflavones and their metabolites with the estrogen receptors (ER) α and β

Compound	ERα	ERβ
GEN	75.4	77.6
G4’G	− 12.4	12.9
G7G	1.2	3.6
G4′7dG	− 13.1	− 35.7
G7S	29.1	14.7
G4′7dS	− 15.0	− 7.1
G7G4’S	0.7	− 27.2
G4’G7S	− 36.7	-55.7
DAI	74.1	76.1
D4’G	− 3.4	16.7
D7G	− 0.3	6.9
D4’,7dG	− 25.2	− 40.6
D4’S	32.9	23.7
D4’,7dS	− 13.5	− 4.3
D7G4’S	1.3	− 29.6
D4’G7S	− 13.3	− 43.5
EQ	73.1	76.9
E7G	− 0.1	19.3
E4’S	32.7	22.3

Note: the higher the score the better the ligand’s fitting into the protein pocket, in agreement with previous studies (Lammi et al. 2020)

**Fig. 11:**
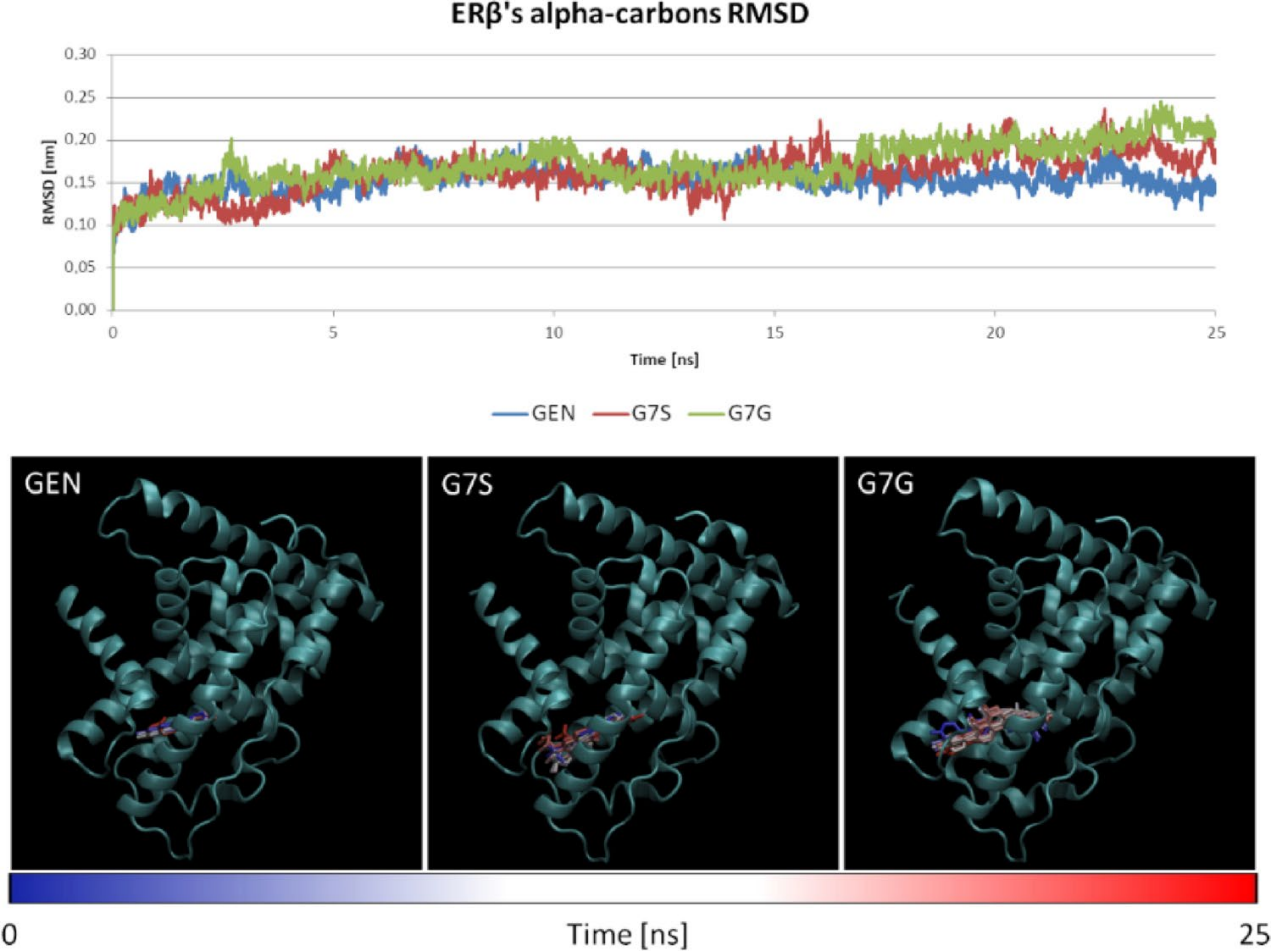
Molecular dynamics results. The protein is represented in cartoon while ligands in sticks. The ligand trajectories are reported in the time-step representation (from red, 0 ns, to blue, 25 ns)

Subsequently, molecular dynamics simulations have been conducted on a selection of ISFs in complex with the ERβ, chosen based on the higher scores the parental compounds recorded therein compared to ERα (that is in line with the higher affinity of phytoestrogens for ERβ previously described (Jiang et al. 2013)). In particular, the analysis was focused on GEN, being the most potent parental compound tested, and its metabolites, which tested significantly active in vitro (i.e. G7G and G7S) to gain insight on the mechanics possibly underpinning their estrogenicity. As shown in Fig. 11, GEN, G7S and G7G were able to stably interact with the ERβ pocket, and the three complexes exhibited a comparable geometrical stability over time as per the analysis of root mean square deviation (RMSD) trend for the alpha carbons of the protein.

## Discussion

Phase II metabolism of substances is considered an inactivation process with an impact on the toxico-/pharmacodynamics of toxic/bioactive compounds. Contrary, some studies have found that ISFs phase II metabolites may contribute to the biological activity of ISFs (Pugazhendhi et al. 2008; Beekmann et al. 2015). Therefore, this study aims to provide a more detailed insight into the role of phase II metabolites in the biological activity of ISFs, specifically focusing on the estrogenicity and cytotoxicity of 16 phase II metabolites of GEN, DAI and EQ in comparison to the parent compounds. The study also investigated whether the results are a direct effect or caused by deconjugation or further metabolization and will deepen the mechanics of estrogenically active compounds through molecular modeling. Human studies consistently show that circulating isoflavones occur predominantly as glucuronide and sulfate conjugates, with free aglycones representing only a minor fraction of total plasma levels. In Western populations, total plasma daidzein and genistein concentrations are typically below 10 nmol/L, while in high soy–consuming populations they generally reach only a few hundred nanomolar (Hüser et al. 2018). Following controlled soy or daidzein intake, plasma concentrations (after enzymatic hydrolysis) can reach the low micromolar range with substantial inter-individual variability (Rüfer et al. 2008). Direct measurements of phase II metabolites further indicate that conjugated forms, particularly daidzein-7-glucuronide-4′-sulfate, constitute the major circulating species, with total metabolite concentrations of approximately 0.6–1.6 µM following soy ingestion (Hosoda et al. 2011). In contrast, under habitual dietary conditions, total serum phytoestrogen concentrations remain substantially lower and generally below 0.1 µM in healthy women (Palma-Duran et al. 2015; Fleck et al. 2016). The estrogenic activities of the substances of interest were investigated from 0.001 to 10 µM in Ishikawa cells expressing both estrogen receptors (ERα and ERβ) utilizing the ALP assay (Johnson et al. 2007). The results showed that all three ISFs exhibited estrogenicity at concentrations greater than 1 μM. However, only DAI showed a slight induction in ALP activity at 0.1 μM. Furthermore, a decrease in estrogenicity at the highest concentration was observed, both were consistent with previous findings (Fig. 2–4) (Grgic et al. 2022).

Interestingly, LC–MS/MS measurements showed that all ISFs underwent sulfation during the 48 h of incubation (Fig. 8–9). For GEN, at 1 µM only 23% non-sulfated substrate could be obtained, while only 6% were metabolized at 10 µM (Fig. 8A/B). The situation looks quite similar for DAI 19% at 1 µM, while higher sulfation with a 69% remaining DAI at 10 µM were observed. In case of EQ, unlike GEN and DAI, the majority of the molecule remained in its aglycone form at both tested concentrations (59% at 1 µM and 85% at 10 µM). The differences between 1 and 10 µM indicate a saturating substrate concentration that reached the maximum reaction rate of sulfotransferases or PAPS (3′-phosphoadenosin-5′-phosphosulfat) depletion at 10 µM, halting further metabolization. This is important as our results suggest that sulfation does not nullify the biological activity, and the previously observed estrogenicity of the aglycones, especially at 1 µM, may be partially attributed to the sulfate-metabolite. However, it would be premature to conclude that sulfation is not a detoxifying pathway without further investigations taking into account the toxicokinetics and, in particular, the clearance profiles of these compounds. Given that ALP expression reaches its peak between 48 and 72 h, and remains stable thereafter, the ALP activity observed is unlikely to result from increased expression during the early hours of incubation (Holinka et al. 1986). Establishing a time event of sulfation might be worth investigating in the future. It is possible that only the aglycones diffuse passively into the cells, exerts their estrogenic effect, and are subsequently metabolized into sulfates, which may be actively transported out of the cells. Further analysis of intracellular metabolites would be needed to clarify these processes.

The sulfation of ISFs is in line with research showing that all four major recombinant human SULTs (SULT1A1, SULT1A3, SULT1E1, SULT2A1) are capable of sulfating GEN and DAI, with SULT1E1 and SULT1A1 exhibiting the highest catalytic efficiency (Nishiyama et al. 2002). SULT1E1 is more active for GEN and DAI with a K_m_ of 0.7 μM and 3.4 µM, respectively, while SULT1A1 has a K_m_ of 0.3 μM and 1.7 µM and the others are significantly higher. While information on EQ being a substrate for SULTs is not available, it seems that it is indeed the case. According to Hevir-Kene and Lanišnik Rižner (2015) SULT1E1 and SULT2B1 are expressed in the Ishikawa cell line, while SULT2A1 was not detected. Another aspect is regioselectivity. Nakano et al. (2004) demonstrated that SULT1E1 catalyzed both 4’- and 7-sulfation of GEN with similar efficiencies, while SULT1A1 showed a clear preference for G7S formation, with a ratio of 8.8. SULT1A1 is regioselective in sulfating DAI, with a preference for the 7-position, while SULT1E1 lacks a regioselective preference, resulting in both sulfate isomers potentially present in the sample . Interestingly, the mRNA expression of SULT1E1 was undetectable in the human breast cancer cell line MCF-7, where only SULT1A1 was present (Fu et al. 2010). In this cell line a previous study found that 4’-sulfation reduced estrogenicity, while 7-sulfation had the opposite effect. The observed difference between Ishikawa and MCF-7 might be explained by the different gene expression between the two cell lines.

In humans, diglucuronides and sulfoglucuronides were found to be major metabolites of GEN in plasma (Soukup et al. 2016a, b). Glucuronidation appears to function as a detoxification process for GEN up to the concentration of 1 μM (Beekmann et al. 2015; Islam et al. 2015). However, at higher concentrations, the glucuronides were found to have estrogenic activity, with G7G showing comparable activity to GEN. The lack of estrogenicity observed for the glucuronides and sulfoglucuronides of DAI is consistent with previous studies, which suggest that glucuronidation acts as a detoxifying process for the aglycone (Islam et al. 2015). Glucuronides have been hypothesized to be unable to alter estrogenic activity due to their size, as they may not fit into ER pockets. Additionally, their hydrophilicity impedes their passage through the cell membrane (Zhang et al. 1999). However, under the current settings glucuronides of GEN were able to induce ALP activity (Fig. 2). At a concentration of 10 μM, G7G exhibited ALP activity comparable to that of GEN. However, it is noteworthy that GEN demonstrated significant estrogenic potential at a much lower concentration of 0.1 μM, indicating its greater potency in eliciting an estrogenic response at lower concentrations compared to G7G. This study also revealed the weak estrogenic potential of G4’,7dG, which was reported for the first time (Fig. 2). Based on the resulting HPLC–MS/MS data (Fig. 8), it can be hypothesized that the estrogenicity at 10 μM is due to the metabolites themselves since none of the compounds were deconjugated to the aglycone. However, further studies examining the processes within the cell are necessary to fully understand the mechanism of estrogenicity at this concentration.

The sulfoglucuronides did not exhibit estrogenic activity at the tested concentrations neither for GEN, nor for DAI. Furthermore, the positioning of functional groups appears to play an important role in the estrogenic activity of the metabolites, with G7S and G4’G showing decreased activity compared to the aglycone (Fig. 2). Overall, these findings suggest that the metabolism of GEN in humans may play a role in reducing its estrogenic activity. It has to be noted that whether the conjugated metabolites are transported into the cells were not assessed in the current study. Due to their increased polarity, conjugates like glucuronides and sulfates typically have limited ability to passively diffuse through cell membranes, unlike their aglycone counterparts. Without determining the intracellular concentrations of these metabolites, it cannot be definitively concluded whether they are inactivated or simply unable to interact with ERs. Furthermore, there are transporters that could potentially facilitate the entry of ISF-conjugates into the cells, which warrants further investigation and ERs located in the membrane and independent of uptake might play a role. These findings should be considered specific to the assay conditions used in this study.

Interestingly, G7S exhibited estrogenic activity at the highest concentration (Fig. 2), suggesting that the sulfate metabolite may be a viable ligand for ERs and the modelling approach showed the highest scores of the GEN-metabolites. However, the absence of G4’S made it impossible to determine whether this result corresponds to one or both isomers, or a combination of both. In case of DAI, the observation that sulfation at the 4’ position shows similar estrogenic effects at 10 µM, contradicts previous studies, which showed that sulfation at this position reduces estrogenicity (Totta et al. 2005; Pugazhendhi et al. 2008). Consistent with this, Kinjo et al. (2004) systematically showed that synthetic daidzein sulfates exhibited minimal estrogenic activity across MCF-7 proliferation assays, ERα/ERβ binding, and ER-dependent reporter gene induction, concluding that hepatic sulfation strongly suppresses intrinsic ER-mediated signaling. In ERβ-only HeLa cells, daidzein-4′-sulfate was inactive across transcriptional and functional endpoints. In contrast, in ERα-dominant MCF-7 cells (ERα:ERβ = 19:1) (Shaw et al. 2006), daidzein-4′-sulfate exhibited measurable estrogenic activity, albeit consistently weaker than daidzein, indicating ERα-dependent partial agonism. Ishikawa cells express both ER subtypes, with ERα positivity in ≥ 95% of cells and ERβ positivity in approximately 56% (ERα:ERβ ratio of 1.6:1) (Zhao et al. 2013), representing an intermediate ER context distinct from both ERβ-only HeLa and ERα-dominant MCF-7 cells. Therefore, the observed differences in activity profiles are best explained by ER subtype composition. LC–MS/MS measurements showed that D4’S, remained unchanged in its structure and was not significantly affected. SULT1E1 has been reported to catalyze disulfation of D7S efficiently (Nakano et al. 2004) . It is important to note that no experiments have been conducted to ascertain whether a metabolite has been broken down to its aglycone and reformed with the functional group occupying a different position in DAI. This discrepancy could potentially explain the differences observed in estrogenicity results when compared to previous studies. There is limited information on whether any of these molecules serve as substrates for enzymes, and to our knowledge, this study represents the first investigation into whether additional metabolism or deconjugation of phase II metabolites takes place in an endometrial cancer cell line. As a metabolite of DAI, EQ undergoes conjugation with sulfates and at 10 µM a similar induction in ALP activity as EQ was obtained (Fig. 4). This potentially results in the retention of biological activity. Furthermore, no cytotoxic effects were observed with the minor exception of dose-dependent proliferative effects for EQ.

In previous experiments EQ was found in urine samples of only 36% of women and 20% of men, while rats and mice were all capable of producing EQ (Soukup et al. 2016a, b). While it was detected in human plasma samples in a previous study, further investigation is required to determine its metabolic processes, tissue distributions, and biological activities. However, none of the two EQ-derivates used in this study showed any changes in ALP activity till the concentration of 0.1 μM, whereas the aglycone presented a 44 ± 27% increase compared to the solvent control (Fig. 4). A higher estrogenicity was observed for 1 μM, reaching 77 ± 13%, followed by 63 ± 29% at the highest concentration of 10 μM. The results of the 1 μM EQ showing the highest induction in ALP activity are in agreement and comparable with previous works (Grgic et al. 2022). Traces of the EQ glucuronide, E7G, have been detected at low concentrations in human plasma (3.5 nM) (Soukup et al. 2014) and represented the second major metabolite according to Obara et al. (2019). Glucuronidation of EQ appears to inactivate the biological ability of the aglycone as no induction of ALP activity was observed for any concentration. Due to the fact that no previous research has been conducted for the effects of E7G on estrogenicity, these results lead to the hypothesis that this might be a detoxification pathway for EQ. That was not the case for the sulfate. Despite showing no changes in estrogenicity for the majority of the concentrations, E4’S depicted a significant increase of 54 ± 8% at 10 µM which was 10% higher than that of EQ at a concentration of 0.1 μM. However, like DAI and D4’S, this effect on estrogenicity has been attributed to sulfation in the 7 position. Sulfation in the 4’ position has been previously shown to reduce, but not fully abolish, the biological activity in MCF-7 cells (Pugazhendhi et al. 2008). Unfortunately, E7S was not available and a comparison was not possible.

The in silico analysis provided deeper insights into the mechanisms behind the estrogenicity of selected ISFs and related metabolites through an integrated approach using docking simulations and molecular dynamics. The docking study revealed that conjugation of ISFs reduced the capability to fit into the agonist–like ERs pocket, in line with previous evidence (Dellafiora et al. 2018b). This reduced fitting capability may correspond to a diminished potential to elicit an agonist-like estrogenic response. In particular, molecules bearing a double conjugation recorded negative scores indicating their substantial incapability to fit into the ERs pocket, in line with the lack of appreciable activity for the di-conjugated GEN and DAI metabolites analyzed in this study. This is likely due to the steric hindrance of di-conjugated metabolites as the pocket of the ER in the active conformation shows a limited capability to arrange bulky and polar ligands (Dellafiora et al. 2018a). Conversely, the presence of mono-conjugation, depending on the position and the ER isoform considered, could allow for fitting the ERs pocket. Of note, G4’G, D4’G, D7G and E7G were predicted fitting in the ERs pocket (i.e. they recorded positive scores), although they were found experimentally inactive. This apparent discrepancy could be explained by the incapability of these metabolites to reach the binding site through the inward ligand pathway, which was not calculated in this study, rather than their actual capability to satisfy the physio-chemical requirements of the pocket.

Moreover, the ability of GEN, G7G and G7S to interact with ERβ over time was calculated through molecular dynamics simulations. The analysis revealed their actual capability to stably persist in the ER binding pocket stabilizing at the same time the agonist-like conformation of the considered ER, as shown by the RMSD analysis. Importantly, the complex with GEN exhibited the most stable RMSD trend providing a convincing explanation for the stronger activity of GEN compared to its conjugated derivatives, as observed in vitro*.* Indeed, the ERβ model for docking and subsequent molecular dynamics was based on the “closed” and agonistic conformation, in agreement with previous studies (Dellafiora et al. 2020), and it is generally assumed that the longer this agonistic conformation is maintained over time, the stronger the agonistic activity (Dellafiora et al. 2020). In this respect, while GEN kept stable the RMSD trend through the entire simulation, G7G and G7S showed a slight increase in the RMSD values from 15 ns onward. This could point to the less stable organization of the agonist organization of ERβ when in complex with G7G and G7S which might be among the mechanics underpinning their lower estrogenic activity. Based on the strong structural similarities among GEN, DAI and EQ sulfates, it can be expected that E4’S and D4’S could stabilize the agonistic conformation as shown for G7S. Additionally, E4’S had a similar induction in ALP activity as EQ at 10 µM (Fig. 4). In the molecular modeling approach, the investigated sulfate metabolites showed clearly positive scores and a better fit to ERα (29.1 to 32.9 units) compared to ERβ (14.7–23.7 units) (Table 1). The hydroxy groups in the 4’ and 7 positions of the phytoestrogens are significant in their interactions with the ligand binding domain (LBD) of ERs, forming hydrogen bonds with different amino acids (Pugazhendhi et al. 2008).

In this study, the estrogenic effects of compounds GEN, DAI, EQ, and 16 phase II metabolites were explored. GEN exhibited significant estrogenic activity at low concentrations, while EQ and DAI showed peak activity at slightly higher concentrations in line with previous studies. Intriguingly, at 1 μM, GEN and DAI were mainly sulfated and these specific metabolites displayed unexpected estrogenic activity, contrary to prior research findings. At the highest concentration (10 µM), all sulfate metabolites were found to be estrogenic, with the order of D4’S > E4’S > G7S These findings challenge the conventional understanding of sulfation as an inactivating process and suggests reevaluation of sulfation and further studies into this issue. Moreover, the study highlighted the importance of considering structural differences in the estrogenic activities of these compounds and their metabolites and emphasize the need for further investigation into the complexities of these interactions. However, ISFs circulate predominantly as phase II conjugates in humans, most of which show little to no estrogenic activity at physiologically relevant concentrations, supporting their largely detoxified role *in vivo*. Although certain sulfate metabolites exhibited activity at 10 µM in vitro, these effects likely reflect mechanistic potential rather than typical exposure levels, which are generally in the nanomolar to low micromolar range. Moreover, rapid in vivo clearance of conjugated metabolites is expected to further limit their systemic biological impact compared with cell culture conditions.

## Supplementary Information

Below is the link to the electronic supplementary material.

Supplementary Material 1

## Data Availability

All data supporting the findings of this study are available within the paper and its Supplementary Information. Should any raw data files be needed they are available from the corresponding author upon reasonable request.
