# Supplementary material for: In vitro evidence for estrogen receptor activity of selected phase II isoflavone metabolites
Source: Arch Toxicol. 2026 Apr 28;100(8):3567–82. doi: 10.1007/s00204-026-04391-2 (PMC13379432; doi:10.1007/s00204-026-04391-2)
Supplement: Supplementary file 1 — Supplementary Material 1 [file 204_2026_4391_MOESM1_ESM.pdf]

Supplementary information of

In vitro evidence for estrogen receptor activity of selected phase II isoflavone metabolites

Dino Grgic[1,2,#], Dimitra Bella-Velidou[1,#], Luca Dellafiora[3], Sebastian T. Soukup[4], Sabine E. Kulling[4], Elisabeth Varga[1,5,*], Doris Marko[1]

[1] Department of Food Chemistry and Toxicology, Faculty of Chemistry, University of Vienna, Währinger Str. 38-40, 1090 Vienna, Austria
[2] Doctoral School in Chemistry, University of Vienna, Währinger Str. 38-40, 1090 Vienna, Austria
[3] Department of Food and Drug, University of Parma, Parma, 43124, Italy.
[4] Department of Safety and Quality of Fruit and Vegetables, Max Rubner-Institut, Federal Research Institute of Nutrition and Food, Haid-und-Neu-Straße 9, 76131 Karlsruhe, Germany
[5] Food Hygiene and Technology, Centre for Food Science, Clinical Department for Farm Animals and Food System Transformation, University of Veterinary Medicine, Vienna, Veterinärplatz 1, 1210 Vienna, Austria

# shared first co-authors
* corresponding author: Elisabeth Varga, elisabeth.varga@vetmeduni.ac.at, +43-125077-3330

Supplementary Table S1: Multiple reaction monitoring parameters of the UHPLC-MS/MS method.

Compound Name	Q1 (*m/z*)	DP (V)	RT (min)	Q3 (*m/z*)	Ratio [*)]	CE (V)	CXP (V)
GEN	268.9	-45	11.1	133.1	-	-44	-15
				159.0	0.42	-42	-21
				131.8	0.36	-48	-11
GEN-4’-glucuronide	444.9	-60	7.8	268.9	-	-34	-17
				113.0	0.39	-22	- 7
				175.0	0.22	-20	-13
GEN-7-glucuronide	444.9	-60	7.2	268.9	-	-34	-17
				113.0	0.52	-22	- 7
				175.0	0.40	-20	-13
GEN-4’,7-diglucuronide	620.8	-105	4.5	268.9	-	-56	-15
				444.8	0.88	-28	-29
				112.9	0.27	-30	-17
GEN-7-sulfate	348.8	-15	9.1	269.0	-	-28	-21
				132.9	0.08	-56	-19
				131.9	0.04	-68	-13
GEN-4’,7-disulfate	428.8	-40	7.5	268.9	-	-36	-17
				348.9	0.69	-22	-23
GEN-7-glucuronide-4’-sulfate	524.8	-20	5.8	269.1	-	-50	-17
				348.7	0.43	-36	-23
				112.9	0.04	-34	-11
GEN-4’-glucuronide-7-sulfate	524.8	-20	6.3	269.1	-	-50	-17
				445.0	0.66	-28	- 3
				112.9	0.10	-34	-11
DAI	252.8	-95	10.2	132.0	-	-50	-13
				223.8	0.99	-36	-15
				208.0	0.97	-42	-13
DAI-4’-glucuronide	428.9	-40	7.1	253.0	-	-34	-17
				112.9	0.59	-20	-15
				174.9	0.33	-18	-15
DAI-7-glucuronide	428.9	-40	6.4	253.0	-	-34	-17
				112.9	0.69	-20	-15
				174.9	0.62	-18	-15
DAI-4’,7-diglucuronide	604.8	-20	3.5	253.0	-	-56	-15
				429.0	0.72	-26	-25
				112.9	0.40	-28	- 7
DAI-4’-sulfate	332.8	-20	8.5 [1)]	253.0	-	-30	-21
				116.9	0.22	-52	-19
				134.8	0.06	-44	- 9

Supplementary Table S1: continued

Compound Name	Q1 (*m/z*)	DP (V)	RT (min)	Q3 (*m/z*)	Ratio *)	CE (V)	CXP (V)
DAI-4’,7-disulfate	412.8	-30	6.9	253.1	-	-38	-19
				332.8	0.91	-18	-23
				224.9	0.08	-64	-19
DAI-7-glucuronide-4’-sulfate	508.8	-30	5.2	253.0	-	-52	-17
				332.9	0.65	-34	-21
				174.7	0.01	-42	-15
DAI-4’-glucuronide-7-sulfate	508.8	-30	5.7	253.0	-	-52	-17
				429.0	0.62	-30	-29
				174.7	0.03	-42	-15
EQ	240.9	-80	10.8	121.1	-	-20	- 9
				118.9	0.72	-26	-13
				134.9	0.47	-24	-19
EQ-7-glucuronide	416.9	-70	7.7	113.0	-	-26	-19
				174.9	0.42	-22	-13
				120.9	0.27	-40	-17
EQ-4’-sulfate	320.8	-60	8.9 2)	121.1	-	-36	-21
				119.0	0.79	-40	-17
				241.0	0.68	-28	-15

The entrance potential (EP) was set to -10 V for all transitions.

Abbreviations: GEN – genistein, DAI – daidzein, EQ – S-equol, Q1 – precursor mass, DP – declustering potential, Q3 – fragment mass, CE – collision energy, CXP – cell exit potential, RT – retention time.

*) The ratio always refers to the first provided transition for each metabolite which was also the one with the highest intensity.

1) The obtained standard does not seem to be as pure as stated since a double peak was visible, see Figure 11. Since no baseline separation was achieved and both compounds were present in the standard the sum of the peak areas was taken into consideration for all the evaluations.

2) No standard for EQ-7-sulfate was available, no clear second peak was visible in the samples, but both isoforms might be present.

Supplementary Table S2: Mean values and standard deviations of recoveries and matrix effects for the four different spiking solutions performed in triplicate.

	Compound	Recovery	Matrix effects
**Isoflavones**	GEN	90 ± 9	88 ± 3
	DAI	82 ± 8	85 ± 1
	EQ	81 ± 6	88 ± 3
**GEN-metabolites**	GEN-4'-glucuronide	86 ± 13	88 ± 5
	GEN-7-glucuronide	91 ± 10	93 ± 3
	GEN-4',7-diglucuronide	79 ± 25	68 ± 10
	GEN-7-sulfate	79 ± 18	91 ± 6
	GEN-4',7-disulfate	86 ± 5	97 ± 9
	GEN-7-glucuronide-4'-sulfate	100 ± 15	98 ± 9
	GEN-4'-glucuronide-7-sulfate	97 ± 12	94 ± 4
**DAI-metabolites**	DAI-4'-glucuronide	86 ± 17	90 ± 7
	DAI-7-glucuronide	97 ± 24	90 ± 2
	DAI-4',7-diglucuronide	<LOQ	<LOQ
	DAI-4'-sulfate	106 ± 7	105 ± 9
	DAI-4',7-disulfate	85 ± 15	72 ± 3
	DAI-7-glucuronide-4'-sulfate	82 ± 25	82 ± 3
	DAI-4'-glucuronide-7-sulfate	87 ± 24	84 ± 3
**EQ-metabolites**	EQ-7-glucuronide	91 ± 1	85 ± 2
	EQ-4'-sulfate	98 ± 18	100 ± 4

Abbreviations: GEN – genistein, DAI – daidzein, EQ – S-equol, < LOQ – below the limit of quantification

Supplementary Table S3: Determined concentrations of the different metabolites after 48 h of incubation (all concentrations provided in nmol/80 µL).

**Products of GEN**	**1 µM [1)]**	**10 µM [2)]**	**Products of DAI**	**1 µM [1)]**	**10 µM [2)]**
GEN	0.02 ± 0.01	0.75 ± 0.44	DAI	0.06 ± 0.01	0.56 ± 0.29
GS	0.07 ± 0.01	0.05 ± 0.03	DS	0.24 ± 0.07	0.26 ± 0.17
**Sum**	0.10 ± 0.02	0.80 ± 0.44	**Sum**	0.29 ± 0.07	0.83 ± 0.34
**Products of G4'G**	**1 µM**	**10 µM [2)]**	**Products of D4'G**	**1 µM**	**10 µM [2)]**
G4'G	n.t.	1.95 ± 1.32	D4'G	n.t.	3.13 ± 0.94
			DS		0.02 ± 0.01
			**Sum**		3.15 ± 0.94
**Products of G7G**	**1 µM [1)]**	**10 µM [2)]**	**Products of D7G**	**1 µM**	**10 µM [2)]**
G7G	0.14 ± 0.4	0.84 ± 0.13	D7G	n.t.	2.22 ± 0.24
GS	0.0007 ± 0.0003	**-**	DS		0.02 ± 0.01
**Sum**	0.14 ± 0.4	**-**	**Sum**		2.25 ± 0.24
**Products of G4',7dG**	**1 µM [1)]**	**10 µM [2)]**	**Products of D4',7dG**	**1 µM**	**10 µM [2)]**
G4',7dG	0.13 ± 0.05	1.22 ± 0.26	D4',7dG	n.t.	0.97 ± 0.31
G4'G	**-**	0.03 ± 0.01	D4'G		0.009 ± 0.002
G7G	**-**	0.02 ± 0.01			
**Sum**	0.13 ± 0.05	1.27 ± 0.26	**Sum**		0.98 ± 0.31
**Products of G7S**	**1 µM**	**10 µM [2)]**	**Products of D4'S**	**1 µM [1)]**	**10 µM [2)]**
G7S	0.07 ± 0.05	1.36 ± 0.14	DS	0.18 ± 0.11	0.67 ± 0.25
**Products of G4'7dS**	**1 µM**	**10 µM [2)]**	**Products of D4',7dS**	**1 µM**	**10 µM [2)]**
G4',7dS	n.t.	1.53 ± 0.29	D4',7dS	n.t.	1.62 ± 0.21
			DS		0.06 ± 0.02
			**Sum**		1.68 ± 0.21
**Products of G7G4'S**	**1 µM**	**10 µM [2)]**	**Products of D7G4'S**	**1 µM**	**10 µM [2)]**
G7G4'S	n.t.	1.90 ± 0.97	D7G4'S	n.t.	1.06 ± 0.22
			DS		0.02 ± 0.01
			**Sum**		1.08 ± 0.22
**Products of G4'G7S**	**1 µM**	**10 µM [2)]**	**Products of D4'G7S**	**1 µM [1)]**	**10 µM [2)]**
G4'G7S	n.t.	1.99 ± 1.11	D4'G7S	n.t.	1.28 ± 0.25
			DS		0.008 ± 0.004
			**Sum**		1.29 ± 0.25

**Products of EQ**	**1 µM [1)]**	**10 µM [2)]**	**Products of E7G**	**1 µM [1)]**	**10 µM [2)]**
EQ	0.02 ± 0.01	0.28 ± 0.22	E7G	n.t.	0.82 ± 0.19
ES	0.02 ± 0.01	0.05 ± 0.04	**Products of E4'S**	**1 µM [nmol/80 µL]**	**10 µM [nmol/80 µL]**
**Sum**	0.04 ± 0.02	0.33 ± 0.22	E4'S	0.09 ± 0.05	0.26 ± 0.12

n.t. – not tested

1) theoretical concentration 0.08 nmol/80 µL

2) theoretical concentration 0.8 nmol/80 µL

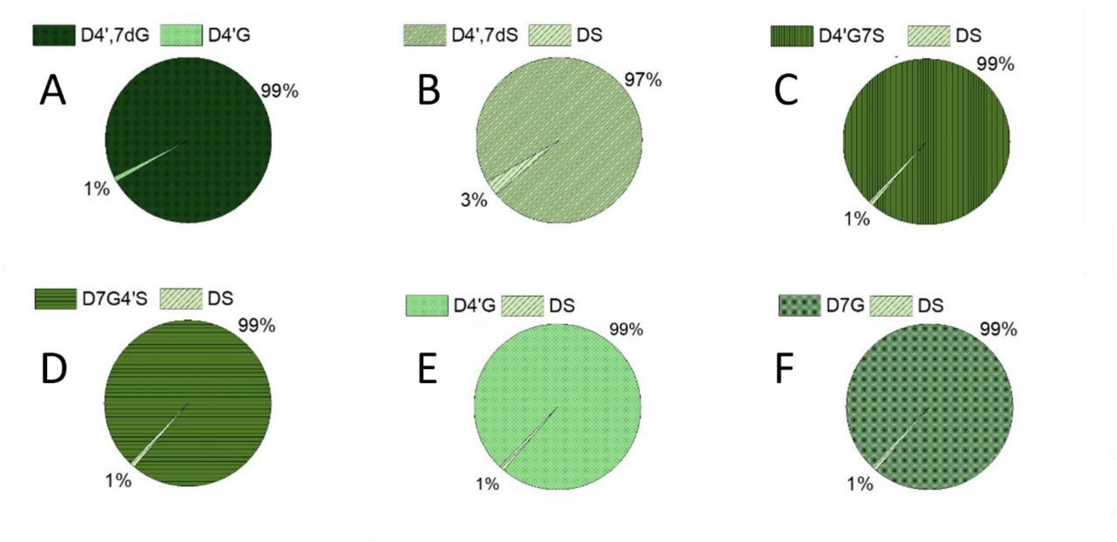

Supplementary Figure S1: Pie charts representing the DAI-metabolites present after incubating Ishikawa cells for 48 h with A - D4',7dG; B – D4',7dS; C – D4'G7S, D – D7G4'S; E – D4'G; F – D7G
